# Professional bodies: how best to promote and support individuals working in human genetics and genomics

**DOI:** 10.1038/s10038-026-01458-x

**Published:** 2026-08-17

**Authors:** William G. Newman

**Affiliations:** The International Federation of Human Genetics Societies, Vienna, Austria

**Keywords:** Social sciences, Health care

Professional bodies are dedicated to the advancement of the knowledge and practice of professions through developing, supporting, regulating and promoting professional standards for technical and ethical competence [1]. They seek to maximise the public benefit of the work undertaken by their members and support the reputation of their professional members. In the UK, the Science Council defines a Professional Body as ‘*an organisation with individual members, practicing a profession or occupation in which the organisation maintains an oversight of the knowledge, skills, conduct and practice of that profession or occupation*.’ Some professional bodies are regulated by the executive at a national level to ensure that professional titles are only used by individuals who are registered and entitled to use these e.g. medical doctors. However, most professional bodies are independent membership organisations that coordinate the activities of a particular profession or a group of closely related professions and represent the interests of their members. It is into this latter group that human genetics and genomics professional bodies fall.

Since the formation of the American Society for Human Genetics in 1948 [2], professional bodies for human genetics have played a fundamental role in setting standards for their members, ensuring quality, and advancing the field through education, ethical guidelines, and advocacy. They facilitate professional development for clinical and laboratory geneticists, genetic counsellors and genetic nurses, scientists and researchers, as well as allied health professionals. They provide resources in genetics and genomics for all healthcare professionals [3] and guide public policy on complex issues like genetic privacy, data use, reproductive technologies, gene therapies, precision medicine [4] and cloning. Their activities include developing educational programs [5], disseminating information on genetic testing, and establishing best practices to support patient care and research [6, 7].

Professional bodies, societies or associations in human genetics and genomics have developed in different geographical regions to act at a national and continental level (see Table 1). Many Societies have seen their primary responsibilities as providing mentorship and training opportunities to the next cadre in the profession; developing new professional roles e.g. bioinformaticians, and promoting advances in knowledge through annual conferences, training programs and public outreach. Efforts to increase access to educational resources through webinars, hybrid conferences and educational apps are all improving the reach and influence of the Societies [5]. Many societies place particular emphasis on supporting the development of genomics clinical services across their continent [8].

**Table 1:** Societies affiliated to the IFHGS

Society	Membership *(Approximate number of members)*	Founded	Website
African Society of Human Genetics (AfSHG)	>1000	2003	https://www.afshg.org
American Society of Human Genetics (ASHG)	8000	1948	https://www.ashg.org
Asia-Pacific Society of Human Genetics	214	2006	https://apshg.info
East Asian Union of Human Genetics Societies (EAUHGS)	9050*	2001	https://www.eauhgs.asia
European Society of Human Genetics (ESHG)	3500	1967	https://www.eshg.org
Human Genetics Society of Australasia	1200	1977	https://www.hgsa.org.au
Latin American Network of Human Genetics Societies (RELAGH)	2500 from 24 societies	2001	https://www.relagh.org
Middle Eastern and North African Medical Genetics Association (MENA-MGA)	1000	2022	https://mena-mga.org

Constituent groups of the International Federation of Human Genetics Societies *6750 in Japanese Society of Human Genetics, 2000 in Chinese Society of Medical Genetics, 300 in Korean Society of Medical Genetics

Recognising the global importance of genomics, and the opportunity for shared learning and coordination of efforts between multi-nation societies to maximise the impact and benefit of genomics, the International Federation of Human Genetics Societies (IFHGS, https://www.ifhgs.org) was formed in 1996 as an umbrella organisation [9]. The Middle Eastern and North African Medical Genetics Association (MENA-MGA) was adopted to membership of the IFHGS in 2025.

The bylaws of the Federation state that:

"*The purpose of The International Federation of Human Genetics Societies (the Federation) is to provide a forum for organized groups dedicated to all aspects of human genetics, including research, clinical practice, and professional and lay education. The Federation will enable communication between its member groups and encourage interaction between workers in genetics fields and in related sciences and will make itself available to promote meetings and publications and other forums which support human genetics research and practice*."

Every five years the IFHGS hosts a congress which brings together members from all continents to discuss genomics, share research, experiences and perspectives of delivering genomic healthcare. The next meeting will be in Guadalajara, Mexico 1-5 March 2027 (https://ic.relagh.org).

Building bridges and forging alliances between the IFHGS and other international institutions is essential for consolidating strategic pathways to progress. In this regard, the Human Genome Organisation (HUGO) plays a key role by bringing together genomics professionals to advance research and education, a mission it has pursued for more than three decades. The Federation is an active member of the HUGO Forum (https://www.hugo-international.org/hugo-forum/).

The maturity, size and resources differ between societies, sometimes reflecting the level of investment in genomics in their countries or regions and the political and public attitudes to genomics. However, many of the ambitions and challenges remain similar.

Professional organisations face challenges in ensuring that the perspective and expertise of their membership is reflected in national debate and policy making relevant to human genomics. To be responsive to the 24/7 news cycle and social media, knowing when to engage in the debate and how to contribute in a constructive manner is difficult, especially when for many societies, leadership is undertaken by individuals on a voluntary basis in addition to their primary professional roles.

Strategic collaboration among human genetics societies is imperative for effective impact. Such concerted action enables the consolidation of clear guidance for their members and, critically, positions the scientific community as an authoritative voice in public and policy discourse. In a context of concerns regarding information integrity, these organizations have a social responsibility to serve as a trusted source of information, providing evidence-based data and expert analysis to the general public and decision-makers. This serves a dual objective: first, to empower the public to form well-founded opinions, and second, to ensure that national, regional and global public policies are built upon a foundation of rigorous scientific knowledge rather than on unsubstantiated claims.

It is vital that Societies act as forums for rich, respectful debate about the future of genomics. Creating opportunities for diverse attitudes and opinions to be heard is essential to ensure that the Societies represent the professions and communities which they endeavour to serve. In addition, it is vital that young members are encouraged to participate and assume leadership roles [10, 11].

We encourage individuals working in human genomics to join their respective national, regional and continental genomics societies, contribute to the leadership, attend conferences to meet colleagues and network working effectively across regions, countries and continents, and ensuring that genomics is made available for the benefit of all.
